# Unraveling the Translational Relevance of β-Hydroxybutyrate as an Intermediate Metabolite and Signaling Molecule

**DOI:** 10.3390/ijms26157362

**Published:** 2025-07-30

**Authors:** Dwifrista Vani Pali, Sujin Kim, Keren Esther Kristina Mantik, Ju-Bi Lee, Chan-Young So, Sohee Moon, Dong-Ho Park, Hyo-Bum Kwak, Ju-Hee Kang

**Affiliations:** 1 Department of Pharmacology, College of Medicine, Inha University, Incheon 22212, Republic of Korea; dwifrista27@inha.edu (D.V.P.); sujin2419@inha.ac.kr (S.K.); kerenmantik@inha.edu (K.E.K.M.); dlwnql@inha.edu (J.-B.L.); thcksdud1@inha.edu (C.-Y.S.); moon219@inha.ac.kr (S.M.); 2 Research Center for Controlling Intercellular Communication, College of Medicine, Inha University, Incheon 22212, Republic of Korea; 3 Program in Biomedical Science and Engineering, Inha University, Incheon 22212, Republic of Korea; dparkosu@inha.ac.kr (D.-H.P.); kwakhb@inha.ac.kr (H.-B.K.); 4 Department of Kinesiology, Inha University, Incheon 22212, Republic of Korea

**Keywords:** β-hydroxybutyrate, lipolysis, sarcopenia, neurodegenerative diseases, inflammation

## Abstract

β-hydroxybutyrate (BHB) is the most abundant ketone body produced during ketosis, a process initiated by glucose depletion and the β-oxidation of fatty acids in hepatocytes. Traditionally recognized as an alternative energy substrate during fasting, caloric restriction, and starvation, BHB has gained attention for its diverse signaling roles in various physiological processes. This review explores the emerging therapeutic potential of BHB in the context of sarcopenia, metabolic disorders, and neurodegenerative diseases. BHB influences gene expression, lipid metabolism, and inflammation through its inhibition of Class I Histone deacetylases (HDACs) and activation of G-protein-coupled receptors (GPCRs), specifically HCAR2 and FFAR3. These actions lead to enhanced mitochondrial function, reduced oxidative stress, and regulation of inflammatory pathways, with implication for muscle maintenance, neuroprotection, and metabolic regulation. Moreover, BHB’s ability to modulate adipose tissue lipolysis and immune responses highlight its broader potential in managing chronic metabolic conditions and aging. While these findings show BHB as a promising therapeutic agent, further research is required to determine optimal dosing strategies, long-term effects, and its translational potential in clinical settings. Understanding BHB’s mechanisms will facilitate its development as a novel therapeutic strategy for multiple organ systems affected by aging and disease.

## 1. Introduction

The human body counts on a complex network of metabolic pathways to maintain homeostasis and respond to alternating energy demands. Among these, ketone bodies emerge as a crucial mechanism for synthesizing alternative energy. Ketone bodies are produced predominantly during states of caloric restriction (CR), prolonged fasting, or exercise. Ketone bodies are precursor and derived lipids that are synthesized by the liver from fatty acids. They are acetoacetate, β-hydroxybutyrate (BHB), and acetone, which are transported to extrahepatic tissues as a fuel in a condition like starvation [1]. BHB, which comprises the largest amount of ketone bodies, not only serves as a vital fuel for peripheral tissues but also has been recognized as promoting resistance to oxidative and inflammatory stress and maintaining metabolic health [2,3]. In addition, BHB triggers a cellular response that adapts by enhancing mitochondrial activity and strengthened mechanisms for combating oxidative stress [4].

Emerging research has highlighted the significance of BHB not only as an energy substrate, but also in various physiological processes of peripheral tissue. An animal study has shown the ability of BHB to enhance the expression of genes associated with mitochondrial biogenesis and the antioxidant defense system in both the liver and skeletal muscles of young rats [5]. In addition, targeting elevated circulating ketones has led to an improvement in heart and skeletal muscle function during cardiometabolic disease [6]. In our previous research, we demonstrated that BHB plays a pivotal role in reducing intracellular lipid accumulation by promoting lipolysis, while simultaneously enhancing thermogenic activity and fat browning, highlighting its potential as a key metabolic [7].

Furthermore, BHB interactions extend to other tissues, such as skeletal muscle and adipose tissue, highlighting a multifaceted network or communication that impacts brain function and overall health. The liver–brain axis provides a critical pathway through which BHB regulates metabolic signaling and influences cognitive health. This includes liver ketogenesis and monocarboxylate-transporter (MCT)-mediated transport for BHB production and its delivery to the brain. BHB also promotes neuroprotection through histone deacetylase (HDAC) inhibition and increased expression of brain-derived neurotropic factor (BDNF). It enhances antioxidant defense by activating nuclear factor erythroid 2-related factor (NRF2). Additionally, BHB exerts anti-inflammatory effects by activating GPR109A and inhibiting the NOD-like receptor family pyrin domain-containing 3 (NLRP3) inflammasome. It supports energy regulation through the activation of AMP-activated protein kinase (AMPK) and stimulation of mitochondrial biogenesis. Finally, BHB contributes to the modulation of peripheral inflammation and the regulation of the hypothalamic-pituitary adrenal (HPA) axis [8,9,10].

In addition, emerging evidence suggest that muscle-derived BHB can enhance cognitive function through myokine signaling, indicating a direct link between skeletal-muscle activity and brain health [11,12]. Similarly, adipose tissue contributes to this communication network by releasing signaling molecules that can affect brain function and energy homeostasis [13]. Understanding this complex connection is important for shedding light on how BHB can contribute to preventing and potentially finding a therapy for disease and promoting cognitive health. In this paper, we will explore an overview of BHB as a ketone body and examine the significance of crosstalk between the liver, muscles, and adipose tissue and the brain.

## 2. Mechanism of Action of β-Hydroxybutyrate

### 2.1. Intermediate Metabolite During Energy Metabolism

It is well known that glucose is considered the primary energy fuel, but during glucose depletion, such as during starvation, calorie restriction, and prolonged exercise, BHB, a major circulating ketone body, steps in as an energy source. BHB is primarily synthesized in the mitochondrial matrix, and its production begins with the condensation of acetyl-coenzyme A (acetyl-CoA) derived from the β-oxidation of fatty acids. Acetyl-CoA generated through this process serves as a precursor for ketone body formation. Ketone bodies, including BHB, are water-soluble and capable of crossing the blood–brain barrier (BBB). Under conditions of excess acetyl-CoA, acetyl-CoA molecules are first condensed by thiolase to form acetyl-CoA. This is followed by the action of 3-hydroxy-3-methylglutaryl-coenzyme A (HMG-CoA) synthase, which catalyzes the condensation of acetoacetyl-CoA with a third acetyl-CoA molecule to form HMG-CoA [14].

**Figure 1:**
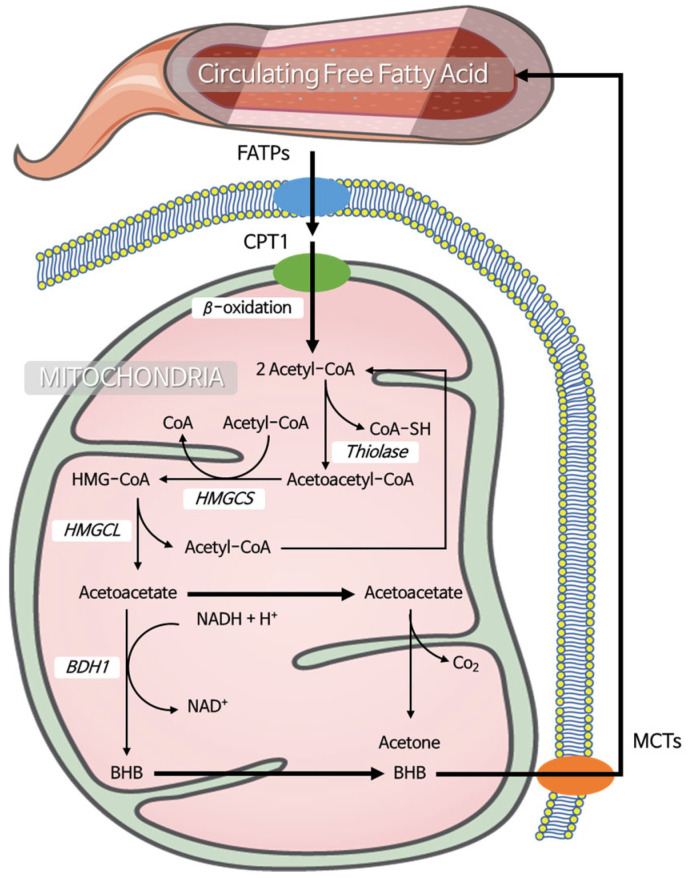
Schematic representation of mitochondrial ketogenesis from circulating free fatty acids. Circulating free fatty acids (FFAs) enter the mitochondria and undergo β-oxidation to generate acetyl-CoA. Two acetyl-CoA molecules are condensed by thiolase to form acetoacetyl-CoA, which is subsequently converted to HMG-CoA by HMGCS (3-hydroxy-3-methylglutaryl-CoA synthase). HMGCL (HMG-CoA lyase) then cleaves HMG-CoA to produce acetoacetate. Acetoacetate can be reduced to β-hydroxybutyrate (BHB) by BDH1 (β-hydroxybutyrate dehydrogenase) or spontaneously decarboxylated to acetone. BHB is then exported from the mitochondria for use as an energy substrate by peripheral tissues.

HMG-CoA plays a dual role. In the cytosol, it is involved in cholesterol synthesis, while in the mitochondria, it participates in ketone body synthesis. The mitochondrial isoform of HMG-CoA is cleaved by HMG-CoA lyase, releasing acetyl-CoA and producing acetoacetate. Acetoacetate can spontaneously degrade to form acetone. Additionally, acetoacetate can be reduced to BHB by β-hydroxybutyrate dehydrogenase, a reaction that depends on the availability of Nicotinamide adenine dinucleotide (NAD^+^) and its reduced form (NADH). NADH then serves as an electron donor to the electron transport chain; however, high elevated NADH levels can inhibit the entry of acetyl-CoA into the tricarboxylic acid (TCA) cycle by shifting metabolic flux toward ketogenesis. Consequently, BHB exits the liver and enters the bloodstream, where it can cross the BBB carried by several monocarboxylic acid transporters, including MCT1 and MCT2, and their expression can regulate BHB uptake and serve as an energy source for the brain and other tissues in need (Figure 1). This metabolic pathway highlights the significance of BHB as an alternative energy substrate during periods of low carbohydrate availability, prolonged exercise, and fasting [15,16].

### 2.2. Signaling Molecule

BHB was once considered solely as a key metabolic intermediate during fasting, prolonged exercise, and the ketogenic state, but now emerging research has recognized it as a multifaceted signaling molecule that bridges energy metabolism with epigenetic regulation; one of the roles is inhibition of HDACs. HDACs are enzymes that play a critical role in epigenetic regulation and cellular homeostasis. A total of 18 HDACs have been discovered in humans, and they are grouped into four classes based on their structural and functional characteristics. HDACs are classified into four classes based on their subcellular localization and cofactor dependency: Zn^2+^. Dependent HDACs are categorized into classes: class I HDACs (HDAC1, HDAC2, HDAC3, HDAC8) are broadly distributed across different tissues and mainly act as gene expression suppressors. Class IIa HDACs (HDAC4, HDAC5, HDAC7, HDAC9) contain only a single catalytic domain. Class IIb HDACs (HDAC6 and HDAC10) are characterized by the presence of two catalytic domains. The class IV HDAC (HDAC11) exhibits high catalytic efficiency, particularly in fatty acid deacetylation. Meanwhile, class III HDACs are NAD^+^—dependent and further divided into four subclasses: subclass I (SIRT1, SIRT2, SIRT3), subclass II (SIRT4), subclass III (SIRT5), and subclass IV (SIRT6 and SIRT17). These subgroups are distributed in different cellular compartments. SIRT1, SIRT6, and SIRT17 are predominantly nuclear, and SIRT 2 is primarily found in the cytoplasm, while SIRT3, SIRT4, and SIRT5 are localized in mitochondria (Table 1) [17,18].

This understanding is essential for developing precise therapeutic strategies, such as class-specific HDAC inhibitors that minimize side effects, and for explaining how metabolic distribution influences aging and degenerative diseases. Therefore, characterizing HDACs by their classes not only release the complexity of cellular regulation but also opens pathways for more effective therapeutic interventions. The crucial role of HDAC inhibition by BHB has been explained by several studies. Shimazu et al. conducted an in vitro experiment using HEK293 cells, treated with BHB at increasing concentrations (1, 2, 4, 8, 16, and 32 mM) that also included TSA (1 uM) and butyrate (4 mM) as comparisons. After an 8 h treatment, they assessed histone acetylation levels via western blot testing, specifically analyzing acetylated histone H3 lysine 9 (AcH3K9) and acetylated histone H3 lysine 14 (AcH3K14). The results showed that BHB enhanced histone acetylation starting from 1 mM, indicating its role as an HDAC inhibitor. They also performed an in vitro assay using immunopurified Flag-tagged HDACs expressed in HEK293 cells. These were immunoprecipitated and incubated with a tritium-labeled acetylated histone H4 peptide in the presence of varying BHB concentrations. The results demonstrated that BHB inhibited HDAC1, HDAC3, and HDAC4 (class I and IIa) with IC50 values of 5.3, 2.4, and 4.5 mM, respectively [19]. A study by Li et al. investigated the role of BHB in regulating microvascular endothelial permeability Claudin-5 expression in human cardiac microvascular endothelial cells (HCMECs). They found that BHB inhibits HDAC3 binding to the Claudin-5 promoter under high glucose (HG) conditions. The study compared HG-stimulated HCMECs with low glucose (LG)-treated cells and tested BHB at concentrations of 1, 3, and 4 mM. While β-catenin levels and its binding to the Claudin-5 promoter remained unchanged across all treatment groups, BHB-treated cells showed reduced HDAC3 binding compared to LG, and HG increased HDAC3 binding. These findings highlight that BHB selectively inhibits HDAC3 recruitment to the Claudin-5 promoter, but β-catenin binding to the promoter remains unaffected, suggesting β-catenin does not play a key role in this regulation under the condition studied [20].

**Table 1:** Classification and biological features of human histone deacetylases (HDACs).

HDAC Class	Members	Cellular Localization	Co-Factor Dependency	Expression	Main Target	Upstream Regulation	References
Class I	HDAC1 HDAC2 HDAC3 HDAC8	Nucleus (HDAC1-2); Nuclear, cytoplasmic, plasma membrane, also mitochondria (HDAC3); Primarily cytoplasmic, also nucleolar (HDAC8)	Zn^2+^	- HDAC 1-3: Broad/ubiquitous, high in proliferative tissues - HDAC 8: Restricted to smooth muscle lineage	- HDAC 1-3: Core histones (H3, H4) at gene promoters, Non-histone proteins: p53, Rb, E2F1, NF-κB, MEF2 - HDAC8: Core histones (H3, H4), Non-histone proteins: SMC3, ERRα, ARID1A, p53, BMF, inv(16), fusion protein, α-tubulin	- HDAC1-2: Sin3, NuRD, coREST, - HDAC2: Nitric oxide, - HDAC3: SMRT/NCoR, - HDAC8: cAMP-CREB1 pathway, TGF-β/SMAD3/4 signaling complex	[21,22,23,24,25,26,27]
Class IIa	HDAC4 HDAC5 HDAC7 HDAC9	Cytoplasm and nucleus (HDAC4); Mainly nuclear, can shuttle to cytoplasm (HDAC5); Nucleus and cytoplasm (HDAC7-9)	Zn^2+^	Tissue-specific; High in skeletal muscle, heart brain, thymus; Alternative splicing HDAC9	Core histones (mainly H3), Non-histone proteins: MEF2, Runx, HIFα, Smad7, NF-kB, MyoD	Phosphorylation Calcium/calmodulin-dependent kinases (CaMKs), protein kinase, D1, cAMP pathway Transcriptional regulation: Sp1/Sp3 (for HDAC4)	[28,29,30,31,32]
Class IIb	HDAC6 HDAC10	Cytoplasmic, can shuttle to nucleus under certain conditions (HDAC6); Mainly cytoplasmic (HDAC10)	Zn^2+^	Heart, skeletal muscle, and brain	Non-histone proteins: α-tubulin, HSP90, cortactin	Phosphorylation (e.g., PKCζ, PKCα, ERK1/2, CK2, GRK2, GSK3β, Aurora A), dynamic protein–protein interactions and cellular stress signals	[33,34,35]
Class III (Sirtuins)	SIRT1-7	Nucleus (SIRT1, SIRT6); Cytoplasm (SIRT2); Mitohondria (SIRT3-SIRT5); Nucleus (SIRT6); Nucleolar (SIRT7)	NAD^+^	Variable: SIRT1/2, broadly expressed in tissues; SIRT3-5, mitochondrial, high in metabolically active tissues; SIRT6, broadly expressed, with notable levels in brain, liver, heart, spleen, adipose tissue, and immune cells; SIRT7, highly expressed in peripheral tissue with high proliferative activity (liver, spleen, testis). Less so in the brain and is found in immune and endothelial cells	Histone deactylation: H1K26, H3K9, H3K14, H3K18, H3K56, H4K6, H4K6, H4K12, H4K16, Non histone: p53, NF-kB RelA/p65 subunit, PGC-1α, FOXO 1/3/4, HSF1, HIF1α, P300, TIP60	AMPK, oxidative stress (conditions that elevate NAD^+^)	[36,37,38,39,40]
Class IV	HDAC11	Nucleus and cytoplasm		Brain, kidney, heart, skeletal muscle	Histone and non-histone proteins (d.g., CDT1); Regulates immune responses by controlling IL-10 expression	Non-coding RNAs (ncRNAs), transcriptional regulations.	[41,42]

Taken together, the inhibition of HDAC class I and IIa at a Zn^2+^-dependent catalytic site by BHB results in histone hyperacetylation, particularly at H3K9/K14 and H4K16 residues in gene-promoter regions. This epigenetic modification regulates the expression of FoxO3a, an anti-aging transcription factor that upregulates MnSOD and catalase, as well as BDNF, a neurotropic gene supporting synaptic plasticity, and metallothionein, which neutralizes free radicals. In addition to its epigenetic role, BHB influences gene expression and cellular responses via other mechanisms, including serving as a metabolic substrate and modulating G-Protein coupled receptors (GPCRs). Specifically, BHB binds to and engages hydroxycarboxylic acid receptor 2 (HCAR2, also known as GPR1O9A) and free fatty acid receptor 3 (FFAR3, or GPR41) [43]. Through HCAR2, a seven-transmembrane GPCR of the Gi family, BHB suppresses the adenylate cyclase/cAMP/protein kinase (PKA) signaling cascade. In macrophages, stimulation of HCAR2 by BHB attenuates inflammasome activity, reduces IL-1β and IL-18 secretion, and inhibits nuclear factor kappa-light-chain-enhancer of activated B cells (NF-kB) signaling, promoting an anti-inflammatory M2 phenotype [44,45,46]. In the brain, HCAR2 activation by BHB modulates immune-cell infiltration, favoring anti-inflammatory macrophage populations, and induces the production of prostaglandin D2 (PGD2) via cyclooxygenase-1 (COX1), contributing to neuroprotection. In the endothelial cells, HCAR2 signaling improves vascular function, while in adipocytes its expression influences lipolysis and supports metabolic adaptation during ketosis [47].

FFAR3 plays a complex role in regulating sympathetic activity and metabolism. While its downstream pathways are not fully elucidated, FFAR3 is highly expressed in sympathetic ganglia and is crucial for sympathetic depression during fasting, which helps conserve energy when nutrients are scarce [48,49]. Unlike short-chain fatty acids (SCFA) that activate FFAR3 by stimulating norepinephrine release, thereby promoting energy expenditure, interestingly BHB has been reported to have conflicting effects on FFAR3. Some studies suggest BHB as antagonist of FFAR3, reducing sympathetic activity and metabolic rate [50]. However, recent research indicated that BHB might actually act as an agonist to FFAR3, highlighting the need for further investigation to resolve this debate. FFAR3 also negatively regulates glucose-stimulated insulin secretion, although BHB’s role in this process appears to be weak [51,52]. The complex interactions between FFAR3, its ligands, and metabolic processes need to be continued to be researched to fully understand its physiological roles.

### 2.3. Muscle–Liver–Adipose-Tissue and Brain Crosstalk During Exercise and Its Relationship with BHB

To fully understand the role of BHB, it is essential to examine the intricate crosstalk between muscle, brain, liver, and adipose tissue, especially during exercise. Physical activity triggers a highly coordinated interplay between these organs, ensuring optimal energy utilization, metabolic flexibility, and adaptive responses to physiological stress. Skeletal muscle, beyond its role in locomotion, functions as an endocrine organ that releases growth factors and cytokines collectively known as myokines, metabolites, and signaling molecules that influence brain function, hepatic metabolism, and adipose-tissue dynamics, especially during exercise [53,54].

The signaling occurs through multiple pathways, primarily mediated by myokines. Some circulating factors, though not all, can cross the BBB and directly interact with brain cells, including irisin, cathepsin B, BDNF, IL-6, and fibroblast growth factor 21 (FGF21). In addition to myokines, muscle-derived metabolites and other non-traditional signaling molecules such as bioactive lipids, enzymes, and exosomes, also play a crucial role in facilitating communication with the central nervous system [54]. During exercise, the motor cortex activates motor neurons to initiate muscle contraction, while the autonomic nervous system regulates energy demands via sympathetic stimulation. Skeletal muscle rapidly consumes adenosine triphosphate (ATP), leading to an increased adenosine monophosphate (AMP)/ATP ratio. This shift activates AMP-activated protein kinase (AMPK), which enhances glucose uptake through GLUT4 (encoded by *SLC2A4*) and promotes fatty-acid oxidation by upregulating carnitine palmitoyltransferase 1 (CPT1, encoded by *CPT1B*). As energy demand rises, muscle glycogen phosphorylase (PYGM) mobilizes glycogen stores, while hexokinase 2 (HK2) catalyzes glucose phosphorylation to drive glycolysis, resulting in pyruvate production. During high-intensity exercise, pyruvate is converted to lactate by lactate dehydrogenase A (LDHA), and exported via monocarboxylate transporter 4 (MCT4, encoded by *SLC16A3*). Lactate is then taken up by the liver through MCT1 (*SLC16A1*) and recycled into glucose via the Cori cycle, involving key gluconeogenic enzymes such as phosphoenolpyruvate carboxykinase (PCK1), glucose-6-phosphate (G6PC), and fructose-1, 6-bisphosphatase (FBP1) [55,56]. At the same time, contracting muscles secrete myokines, such as IL-6 (encoded by *IL6*), irisin (from FNDC5), and BDNF, which exert systemic effects. IL-6 acting via JAK/STAT3 signaling stimulates hepatic glucose production and enhances fatty-acid oxidation by increasing PPARα expression. Irisin, cleaved from FNDC5, crosses the blood–brain barrier (BBB) and upregulates BDNF in the hippocampus, promoting synaptic plasticity and neurogenesis. Simultaneously, adiponectin (encoded by *ADIPOQ*), primarily secreted by adipose tissue, increases fatty-acid oxidation in muscles (via AMPK and PGC-1α/PPARGC1A activation), improving endurance [57].

Meanwhile, sympathetic activation stimulates adipose tissue to increase lipolysis through β-adrenergic receptors (ADRB1/2/3), leading to the breakdown of triglycerides by hormone-sensitive lipase (HSL) and adipose triglyceride lipase, releasing free fatty acids (FFAs) into circulation. These FFAs are taken up by muscle via CD36/FATP transporters and undergo β-oxidation in mitochondria [58]. In prolonged exercise, as glycogen stores deplete, the liver shifts to ketogenesis by upregulating 3-hydroxy-3-methylglutaryl-CoA synthase 2 (HMGCS2) and β-hydroxybutyrate dehydrogenase (BDH1), producing BHB, which is alternative energy source for both the brain and muscle [59,60].

In the brain, BHB inhibits HDACs (particularly class I and IIa) enhancing the transcription of neuroprotective genes (FOXo3a, SOD2, and catalase/CAT), which mitigate oxidative stress [61]. BHB also suppresses neuroinflammation by inhibiting the NLRP3 inflammasome, reducing the release of pro-inflammatory cytokines (TNF-α, IL-1β, IL-18) by microglia [62]. Additionally, NF-kB (encoded by RELA/NFKB1) deacetylation is inhibited by BHB, reducing systemic inflammation while increasing TP53 acetylation, promoting cellular repair and apoptosis in damaged cells [62,63].

**Figure 2:**
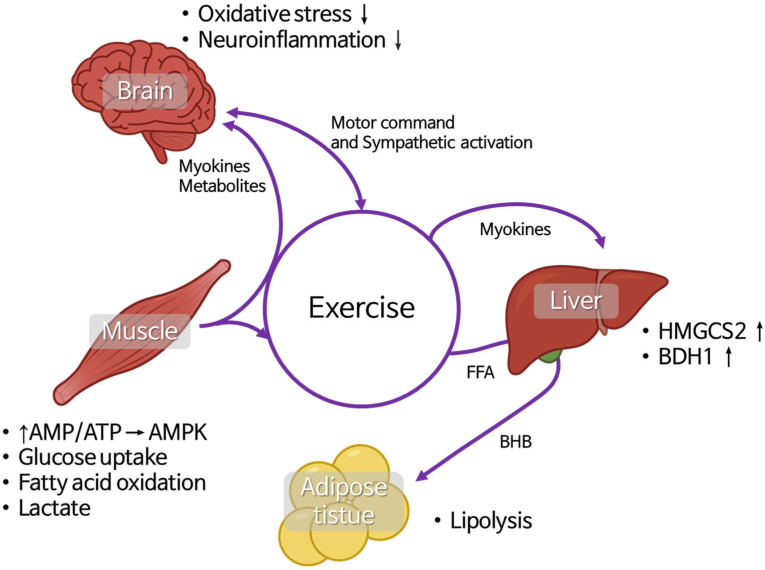
Inter-organ metabolic interactions during ketogenesis induced by exercise. During periods of prolonged exercise, enhanced lipolysis in adipose tissue increases circulating free fatty acids (FFAs), which are taken up by the liver and converted into ketone bodies, primarily β-hydroxybutyrate (BHB). Skeletal muscle utilizes BHB for ATP production, especially when glucose availability is limited. In parallel, the brain adapts to utilize BHB as a major fuel source, preserving glucose for essential biosynthetic pathways. During exercise, high-intensity in particular, myokines released from skeletal muscle and metabolites such as BHB and lactate play roles in dynamic metabolic crosstalk between peripheral tissues and brain in states of ketone-body production.

As exercise progresses, the interplay between brain, muscle, liver, and adipose tissue ensures energy balance, mitochondrial efficiency, reduction of inflammation and neuroprotection, optimizing both physical performance and cognitive function (Figure 2).

### 2.4. Circadian Regulation of BHB Metabolism and Signaling

Like many physiological processes, the metabolism and signaling functions of BHB are regulated by the circadian clock. In mammals, energy metabolism is tightly orchestrated by this internal timing system to align physiological processes with feeding–fasting cycles. The central circadian pacemaker, located in the suprachiasmatic nucleus, synchronizes peripheral clocks in metabolic organs such as the liver, skeletal muscle, and adipose tissue. These clocks coordinate daily rhythm in nutrient sensing, hormone secretion, mitochondrial activity, and substrate utilization in a time-of-day-dependent manner. The liver, as the primary site of ketogenesis, displays pronounced circadian rhythmicity in the expression of key enzymes, including HMGCS2 and BDH1, which are directly involved in BHB synthesis [64,65,66].

Recent findings by Mezhnina et al. provide compelling evidence that hepatic ketogenesis is under direct transcriptional control by the circadian clock machinery. Using chromatin immunoprecipitation assays, they demonstrated that BMAL1 and CLOCK, the core components of the molecular clock, bind to the promoter regions of *HMGCS2* and *BDH1*, driving their rhythmic expression. The expression of these genes, along with the normal rhythmic production of BHB, peak in a time-dependent manner. These findings highlight the essential role of BMAL1 in the temporal regulations of ketogenesis and suggest that circadian disruption may impair BHB synthesis and reduce metabolic flexibility [67].

In addition to metabolic regulation, BHB also exerts epigenetic effects, particularly through HDAC inhibition. HDAC activity itself is modulated in a circadian manner. For instance, HDAC3 demonstrates rhythmic recruitment to chromatin in hepatocytes, guided by the nuclear receptor Rev-erbα, which is also a core circadian output. Similarly, sirtuins, particularly SIRT1, are both regulated by the circadian clock and influence circadian rhythms by deacetylating CLOCK and BMAL1, thereby affecting circadian gene expression [68,69].

Importantly, circadian disruption—due to factors such as shift work, sleep deprivation, or irregular eating patterns—has been shown to impair HDAC and SIRT activity, disrupt BHB production, and predispose individuals to metabolic diseases, including non-alcoholic fatty liver disease, non-alcoholic steatohepatitis, and Type 2 Diabetes Mellitus (T2D). In such states, the time-of-day-specific responsiveness to BHB may be blunted, reducing its anti-inflammatory and epigenetic benefits. Altogether, these findings support the idea that understanding not only how BHB levels can be elevated, but also when they are endogenously produced or therapeutically administered, is essential. Incorporating the circadian dimension may enhance the precision of BHB-related interventions for metabolic, inflammatory, and age-related diseases [68,70,71].

## 3. Role of β-Hydroxybutyrate in Diseases

### 3.1. Sarcopenia

Sarcopenia, characterized by the progressive loss of skeletal muscle mass, strength, and function, is a major contributor to frailty and reduced quality of life. While aging is the primary underlying cause, sarcopenia can also result from various disease-related risk factors that promote muscle wasting and atrophy, including bone and joint disorder, cancer, and chronic conditions such as cachexia. Additionally, sarcopenia is frequently associated with prolonged hospital stays, malnutrition, and immobility [72,73]. A recent study by Lin et al. demonstrated how BHB mitigates stroke-related sarcopenia by using a transient middle cerebral artery occlusion mouse model. Mice were treated subcutaneously with either 5 mg/kg BHB or 6 mg/kg of glucose for 3 and 7 days. The results showed that BHB-treated mice exhibited improved paretic extensor digitorum longus (EDL) muscle mass after a stroke compared to the glucose-treated group. Furthermore, the study revealed that BHB directly binds to the FOXO3a promoter and the histone mark H3K9, suggesting an epigenetic mechanism underlying its protective effects [74]. As previously discussed regarding BHB as a signaling molecule, this aligns with the evidence demonstrating that BHB induces H3K9 histone lysine β-hydroxybutyrylation (Kbhb), thereby facilitating FOXO3a binding to DNA and promoting longevity-associated gene expression.

Emerging evidence highlights the therapeutic potential of BHB in combating sarcopenia through its effects on mitochondrial health. Wang et al. demonstrated that BHB reverses age-related muscle atrophy and improves motor function in both C. elegans and mouse models. This effect was mediated, in part, by BHB-induced Kbhb, which upregulated genes involved in oxidative phosphorylation, ATP production, and mitochondrial biogenesis [75]. Complementing these findings, Parker et al. investigated the direct impact of BHB on skeletal muscle mitochondria and found that BHB enhances mitochondrial efficiency by reducing hydrogen peroxide (H_2_O_2_) emission and mitochondrial fission, potentially via suppression of ceramide accumulation. Notably, these mitochondrial benefits occurred without increasing mitochondrial number, suggesting improved function rather than quantity [76]. Together, these studies suggest that BHB not only acts as an epigenetic regulator but also enhances mitochondrial resilience and redox balance in skeletal muscle, supporting its potential as a therapeutic strategy for age-related muscle degeneration such as sarcopenia.

### 3.2. Cancer and Cachexia

Cancer is a complex disease characterized by uncontrolled cell proliferation, metabolic programming, and resistance to apoptosis. Recent studies suggest that BHB plays a multifaceted role in cancer progression and therapy. Traditionally recognized as an alternative energy substrate, BHB has gained attention for its impact on cellular metabolism, epigenetic regulation, and the inflammatory pathway, all of which are critical in tumorigenesis [77].

BHB has the ability to modulate histone acetylation via the inhibition of HDACs, particularly class I and IIa HDACs, leading to the enhanced expression of tumor-suppressor genes, such as *FOXO3a* and *TP53*, which promote apoptosis and cellular repair. Additionally, BHB has been shown to reduce NF-kB signaling, suppressing pro-inflammatory cytokines like TNF-α, IL-6, and IL-β, which are commonly elevated in the tumor microenvironment [78,79]. Moreover, BHB affects cancer metabolism by inhibiting glycolysis and shifting cancer cells toward oxidative phosphorylation, which may limit their proliferative potential [80].

However, the role of BHB in cancer is context-dependent. While it exhibits tumor-suppressive effects in certain cancers, some studies suggest that BHB may serve as an energy source for highly oxidative tumors, potentially supporting their growth [81,82,83,84,85]. Therefore, understanding the molecular mechanism and metabolic dependencies of different cancer types is crucial for evaluating the therapeutic potential of BHB in oncology (Table 2).

Cancer cachexia occurs depending on the type and stage of cancer and is responsible for approximately 20% of all cancer-related deaths. Among the major components of cachexia is muscle-wasting-induced sarcopenia, which is driven by enhanced catabolic metabolism mediated by inflammatory cytokines released from cancer cells. This process has a detrimental impact on cancer prognosis [86]. Recently, there has been growing interest in the effects of ketones on cachexia, and some studies have reported that BHB may exert anti-catabolic effects in cancer cachexia [87]. BHB has also been shown to suppress inflammation and oxidative stress, which are key pathogenic mechanisms of cancer cachexia. However, one study reported that, despite achieving elevated blood BHB levels through a ketogenic diet, there was no beneficial effect on the prevention of cachexia or reduction in mortality in a lung-cancer-induced cachexia mouse model [88]. Therefore, further research is needed to clarify the mechanisms and efficacy of BHB in the suppression of cancer cachexia.

### 3.3. Diabetes Mellitus

Diabetes mellitus (DM) is metabolic disorder presenting with a high blood-glucose level (hyperglycemia), polyphagia, and polydipsia, and it results from either an autoimmune process that leads to the destruction of pancreatic β cells resulting in absolute insulin deficiency in type 1, or, in contrast, type 2 DM, which involves a combination of insulin resistance and a relative insulin deficiency [89]. DM is known as one of the fastest growing diseases worldwide. Several factors contributing to the increasing prevalence of DM type 2 are obesity, a modern lifestyle characterized by high-carbohydrate and high-sugar diets, increased consumption of processed food containing preservatives, and a sedentary lifestyle with a lack of physical activity [90,91,92]. DM, if left untreated or treated poorly, causes not only well-known complications such as coronary heart disease, stroke, peripheral neuropathy, diabetic ulcers, diabetic kidney disease, and retinopathy but also emerging complications that have been reported, such as cancer, infection, liver disease, affective disorder, and functional disability, as well as cognitive disability that leads to high morbidity and mortality [93,94,95].

An in vitro and in vivo study by Zhang et al. revealed significant findings on the therapeutic potential of BHB for type 2 DM. They found that BHB ameliorates insulin resistance in T2D mice through a mechanism involving the HCAR2/Ca^2+^/cAMP/PKA/Raf/ERK1/2/PPARγ pathway. They demonstrated that exogenous BHB supplementation via 1,3-butanediol (1,3-BDO) drinking solution could safely raise blood BHB levels without causing ketoacidosis. At physiological concentrations, BHB binds to HCAR2, leading to an increase in intracellular Ca^2+^, activation of adenylyl cyclase, and subsequent elevation of cAMP/PKA activity. This signaling cascade promotes ERK-dependent, rather than CDK5/p25-dependent, phosphorylation and transcriptional activation of PPARγ, thereby enhancing the expression of genes associated with insulin sensitivity. These effects contribute to the modulation of insulin signaling and may underlie the therapeutic potential of BHB in T2D [96]. Another study by Jung et al., both in an in vitro and in vivo experiment, revealed that BHB has protective effects against diabetic nephropathy (DN) through increasing autophagy in renal proximal tubule cell line HK-2 cells. BHB treatment increased LC3I and LC3II ratios in non-serum starved HK-2 cells at 2 and 8 h and additionally increased the phosphorylation level of AMPK and beclin, reducing the level of p62, enhancing NRF2 expression. Additionally, BHB reduced high-glucose induced reactive oxygen species (ROS) levels in these cells. The in vivo study verified these findings, showing increased LC3 and decreased ROS levels in the kidney of mice on a ketogenic diet [97]. These findings suggest that BHB has potential as a therapeutic agent improving various aspect of diabetes, including insulin sensitivity, glucose regulation, and protection against complications such as diabetic neuropathy.

A recent study by Neudorf et al. investigated the anti-inflammatory effects of BHB in humans, focusing on individuals at risk of developing T2D. They found that ex vivo application of BHB at a concentration of 2–10 mM to immune cells produced acute anti-inflammatory effects, characterized by suppression of pro-inflammatory cytokines (TNF-α. IL-1β, IL-6) and increased expression of anti-inflammatory cytokines such as IL-10 and IL-1 receptor agonist. These findings suggest a potential protective role of BHB against inflammation-driven insulin resistance. However, they suggested that further studies are needed to elucidate the effects of BHB administration under different conditions, including disease status, glycemic levels, and duration of exposure. Specifically, in individuals with established T2D, short-term elevation of BHB levels via ketone monoester supplementation transiently increased plasma IL-10 levels; however, this effect was not sustained after 14 days of repeated treatment. In addition, chronic hyperglycemia appeared to diminish BHB’s anti-inflammatory potential, reducing its ability to suppress TNF-α and IL-1β expression in immune cells. Although direct ex vivo treatment with BHB continued to modulate cytokine secretion, the effect was less stable and less pronounced compared to that observed in at-risk individuals [98]. These findings suggest that the anti-inflammatory effects of BHB are both time-sensitive and context-dependent. While BHB appears acutely beneficial in individuals at risk of T2D, its efficacy diminishes established T2D, potentially due to chronic hyperglycemia. This highlights the possibility that continuous BHB supplementation, rather than intermittent intake, may be required to maintain its therapeutic effects in diabetic patients.

### 3.4. Cardiovascular Disease

The myocardium is a high ketone-body consumer, especially when other substrates are limited, and increased myocardial delivery and oxidation of ketone bodies occur in patients with advanced heart failure. Increasing BHB levels before ischemia/reperfusion injury reduces infarct size in rodents, and exogenous BHB delivery can protect the heart from ischemia/reperfusion injury (I/R injury), reduce mitochondrial ROS formation, and increase ATP production. Moderately elevated BHB is cardioprotective during I/R and heart failure as it enhances energetic homeostasis. BHB acts as an endogenous NLRP3 inflammasome inhibitor, reducing inflammatory responses, and increasing circulating BHB levels may treat NLRP3 inflammasome-related cardiovascular diseases [99].

A recent study by Li et al. using C57BL6 male mice with myocardial infarction (MI) found that the serum BHB levels rise up to 1 to 2 mM after 2 weeks of ketone ester supplementation. Echocardiographic analysis showed that mice treated with BHB improved cardiac function, reduced infarct size and fibrosis, and increased vascular density. In addition, the in vivo as well as in vitro study showed BHB increased H3K9bhb levels upregulating carnitine palmitoyl transferase 1A (*CPT1A*) and related genes. The *CPT1A* gene itself contributes to angiogenesis via blood-vessel remodeling and fatty acid oxidation in the hypoxic condition [100]. The beneficial role of CPT1A upregulation in cardiomyocytes was also supported by another study in which knockdown of *CPT1A* suppressed the growth of cardiomyocytes also under the condition of chronic hypoxia [101]. Another in vivo study also showed results of the lipid profile after BHB treatment increased high density lipoprotein (HDL) levels and decreased total cholesterol (TC); low-density lipoprotein (LDL) and triglyceride (TG) levels in mice during the glycemic response were also improved. The study also found out that male Apo -/- mice fed with a Western diet plus treatment with BHB 100 mg/kg by oral gavage daily for 24 weeks have lower aortic calcium content as well as lower alkaline phosphatase (ALP) activity in the aorta compared to control mice. Furthermore they showed that BHB reduced ALP activity and calcium deposition in vascular smooth muscle cells (VSMCs) thus inhibiting VSMC calcification in vitro [102]. Atherosclerotic calcification results from calcium phosphate crystallization, and even small crystals can contribute to the formation of atherosclerotic plaques by inducing inflammation and leading to VSMC death. Notably, atherosclerotic calcification is a significant risk factor for cardiovascular disease, particularly myocardial infarction (MI) [103], These findings suggest that BHB treatment may have a protective effect by reducing the risk of MI. A recent study has found that HDAC9 is a key regulator and could be a potential therapeutic target for VSMC-driven cardiovascular disease [104]. As previously mentioned, BHB functions as an epigenetic modifier and inhibits HDACs, particularly those belonging to class I and IIa. Notably, HDAC9 falls within in the class IIa subgroup [105,106]; this aligns with the idea that BHB-mediated HDAC inhibition may contribute to its cardioprotective effects.

## 4. Lifestyle Interventions to Enhance β-Hydroxybutyrate Levels

BHB levels can be regulated through various interventions. One of the most effective natural methods to increase BHB levels is adherence to the ketogenic diet (KD). The KD is a dietary plan with very low carbohydrate, high fat, and moderate protein intake. It is designed to induce a metabolic state that mimics fasting, thereby promoting ketogenesis without the adverse effects associated with prolonged starvation. It typically follows a specific macronutrient distribution of fats comprising the majority of caloric intake. Ranging from 60% to 90% of total energy, with a 70–75% common target, carbohydrates are severely restricted, usually limited to less than 50 g per day. This translates to approximately 5–10% of total caloric intake. Meanwhile the protein intake is moderate, calculated at 1–1.7 g per kilogram of body weight. This generally counts for about 20% of the diet’s total energy value [107]. This macronutrient composition—characterized by a high-fat and restricted carbohydrate intake—is intentionally formulated to induce and maintain a state of nutritional ketosis. In this metabolic state, the body shifts from using carbohydrates as its primary fuel source to relying predominantly on fat. As a result, the liver initiates ketogenesis, a process that produces ketone bodies such as BHB [108,109]. The KD has long been recognized as an effective non-pharmacological intervention for patients with epilepsy. In recent years, despite debates, a growing body of research has also suggested that the KD may offer potential therapeutic benefits for various neurodegenerative diseases [110,111].

A study by Jarett et al. confirmed that the KD increases mitochondrial glutathione levels in adolescent Sparague–Dawley rats. The rats were fed either a KD or a control diet for three weeks. Ketosis was monitored by measuring serum BHB levels weekly. BHB concentrations were significantly higher in the KD group compared to the control group after just one week and reached a steady state by the second week. Additionally, blood glucose levels were significantly lower in the KD group after three weeks. Their study also found the neuroprotective effects of the KD through promoting glutathione (GSH) biosynthesis, strengthening mitochondrial antioxidant defenses, and the protection of mitochondrial DNA (mtDNA) from oxidative damage. Specifically, the KD enhances de novo synthesis of mitochondrial GSH and improves redox balance in the hippocampus, leading to reduced mitochondrial ROS production and protection of mtDNA [112].

Another benefit of the KD for neuroinflammation is demonstrated in a study by Polito et al., in which BHB, a product of the KD, acts as an anti-inflammatory agent in microglial cells (BV2). The study found that BHB promotes M2 polarization, reduces migration in response to lipopolysaccharide (LPS)-induced inflammation, and modulates cytokine levels by decreasing IL-17 and increasing IL-10. These findings suggest that increasing BHB levels through the KD may play a key role in modulating neuroinflammation and could contribute to neuroprotection, with potential benefits in preventing neurodegenerative diseases [113]. However, further research is needed to fully understand the underlying molecular mechanism.

Interestingly, the KD has also been reported to have a beneficial effect on maintaining skeletal muscle. Wallace et al. showed that the KD can effectively alleviate sarcopenia in aging mice. Their study demonstrated that mice on a KD maintained greater skeletal muscle mass compared to those on a control diet. This effect was associated with a shift in muscle fiber type, from IIb to IIa, along with enhanced mitochondrial biogenesis, oxidative metabolism, and antioxidant capacity, all of which contribute to a healthier cellular environment. In addition, the KD reduced endoplasmic reticulum (ER) stress, protein synthesis, and proteasome activity, suggesting a decrease in protein turnover and a reduction in overall cellular stress [114].

Emerging clinical evidence suggests that KD may offer supportive benefits for patients with advanced cancer. Several studies have reported improved outcomes associated with KD interventions. For example, Schimdt et al. found that patients who completed a three-month KD intervention experienced improved emotional functioning and reduced insomnia. Although some individuals reported temporary side effects such as constipation and fatigue, no severe adverse events occurred [115]. In another study, Egashira et al. observed that patients on a chronic KD regimen (>12 months) had better overall survival rates compared to those who followed the diet for a shorter duration (<12 months), indicating that the length of adherence may be a critical factor in the therapeutic effectiveness of the KD in cancer care [116].

Despite the benefits of the KD, several side effects have been reported, including constipation, diarrhea, muscle cramps, headache, halitosis, and nephrolithiasis [117]. These potential complications highlight the need for careful monitoring, particularly in long-term use, to ensure safety and minimize health risks.

Additionally, case reports have documented severe elevations in TC, LDL, and TG in individuals following the KD, even in those without pre-existing hyperlipidemia, suggesting that the diet may exacerbate or unmask underlying lipid disorders. These findings emphasize the importance of individualized risk assessment and routine lipid monitoring for individuals on a KD, especially those with a family history of cardiovascular disease or dyslipidemia [118].

Fasting is another effective strategy to elevate circulating BHB levels, as mentioned before. During prolonged fasting or intermittent fasting protocols, the body shifts from glucose metabolism to fatty-acid oxidation, leading to hepatic ketogenesis and an increase in ketone bodies, particularly BHB. This metabolic shift not only provides an alternative energy source for peripheral tissues and the brain but also has been associated with anti-inflammatory effects, improved metabolic flexibility, and cellular stress resistance [119]. Several studies have reported that both intermittent and time-restricted fasting can significantly raise BHB concentrations, suggesting their potential utility in therapeutic contexts. For example, alternate-day fasting (ADF), a specific form of intermittent fasting, has been shown by García-Juárez et al. to elevate plasma BHB levels and exert neuroprotective effects. In a model mouse with autism-like behavior induced by prenatal exposure to a cafeteria diet, ADF increased anti-inflammatory CD206^+^ microglia in the hippocampus, reduced ER stress markers (BiP, ATF6, p-JNK), and improved social interaction. This modulated neuroinflammation and microglial complexity, contributing to improved neurobehavioral outcomes [120].

A growing body of evidence highlights that ketone responses to fasting vary significantly with age. A recent pediatric study explored fasting-induced BHB levels in healthy children and found that younger participants, particularly those under the age of three, displayed a greater tendency to produce ketones during short fasting periods. Despite this age-related variability, elevated BHB levels above 1.0 mM were infrequent across the cohort. These findings point to the potential of using fasting BHB thresholds to help flag atypical ketone responses, which may reflect underlying metabolic vulnerabilities. Defining age-appropriate reference ranges for BHB could thus improve early screening and differentiation between physiological adaptation and hidden pathological conditions in children prone to hypoglycemia [121]. Importantly, Berger et al. demonstrated that even individuals with type 1 diabetes (T1D), a population typically considered vulnerable to fasting, can tolerate a seven-day fasting protocol safely. In their pilot study, adults with T1D achieved mean BHB levels of 2.9 ±1.9 mM by day 7 while maintaining stable blood glucose and significantly reducing insulin requirements. Improvements were also noted in quality-of-life scores, body weight, and BMI, with only temporary and manageable side effects [122]. These findings further support the feasibility of the metabolic benefits of fasting as a means to safely elevate systemic BHB, even in metabolically vulnerable populations.

**Figure 3:**
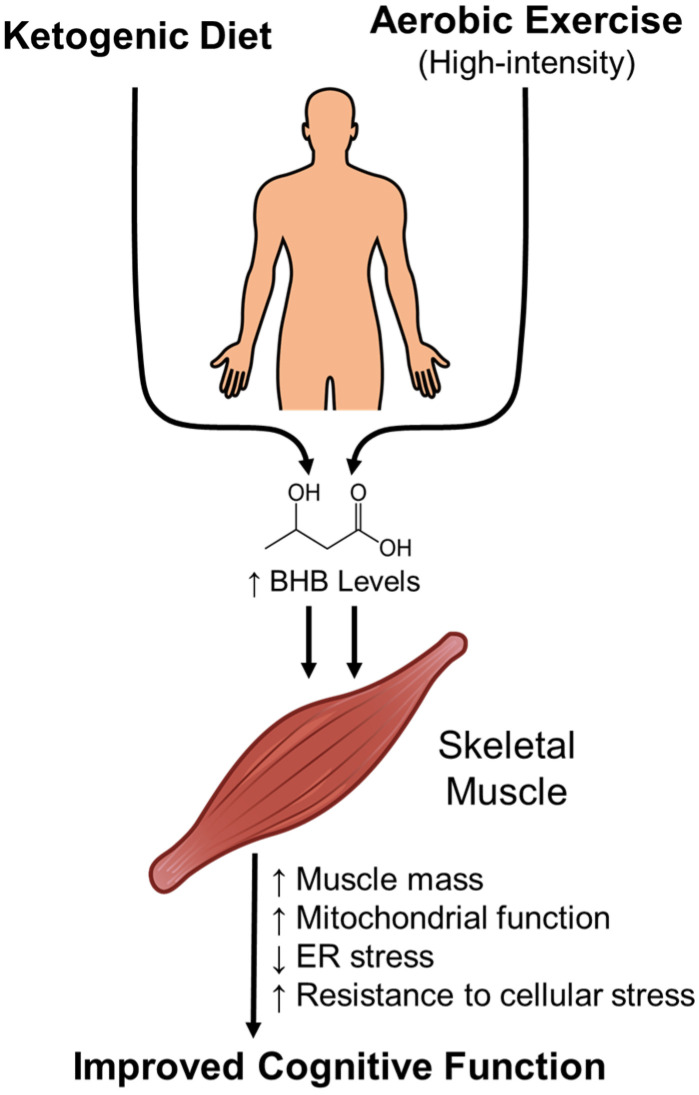
Proposed effects of ketogenic diet and exercise-induced BHB elevation on skeletal muscle and cognitive function. The ketogenic diet and exercise, high-intensity in particular, both elevate circulating BHB level. Elevated BHB is associated with skeletal muscle health through increased muscle mass, mitochondrial biogenesis and oxidative metabolism, and reduced cellular stress. Recent evidence suggests that exercise-induced BHB elevation may also support cognitive function, particularly in individuals with low baseline BHB levels. Together, these findings highlight the potential of BHB as a metabolic mediator linking lifestyle intervention with musculoskeletal and neurologic benefits.

Our previous study in rats has shown that both exercise and the KD can elevate circulating BHB levels. The KD alone increased the mean plasma BHB concentration to approximately 1 mM, while exercise, high-intensity in particular, further enhanced BHB levels, doubling those observed in control groups fed a normal diet. One hour after exercise, the mean plasma BHB levels were 0.8 mM in the exercise group and 1.6 mM in the combined exercise and KD group [7]. Notably, a recent study by Kwak et al. suggests that aging reduces BHB levels, but endurance exercise can counteract this decline by promoting ketone production. In aged mice, endurance exercise increased serum and skeletal muscle BHB levels, whereas this was not the case for resistance exercise. Similarly, in elderly women who completed a 16-week exercise program, BHB levels were positively correlated with skeletal-muscle function, indicating a potential role in muscle health. Initially, no clear link was found between exercise-induced BHB and cognitive function, but after excluding individuals with naturally high BHB levels, a positive association emerged; this proposed that exercise-induced BHB release may enhance both skeletal muscle and cognitive function, especially in individuals with lower baseline BHB levels [123]. Therefore, the increase in BHB levels induced by the KD and exercise may contribute to the maintenance of skeletal muscle health and cognitive function (Figure 3). Overall, it should be noted that exercise, the KD, or BHB’s supplementation interventions aimed at increasing BHB levels should be personalized and carefully monitored, as individual health status, metabolic responses, and underlying conditions can vary significantly (Table 3).

## 5. Potential Therapeutic Applications of β-Hydroxybutyrate

Exogenous ketone supplements, such as Medium chain triglycerides (MCT), Ketone esters (KE), and D-β-hydroxybutyrate (D-BHB), are increasingly used as alternatives to the KD to induce ketosis without requiring dietary restrictions. MCTs, composed of fatty acids, are rapidly metabolized in the liver. While high doses of MCT can moderately increase blood ketone levels by 0.5–1 mM, their use is often limited by gastrointestinal intolerance and a high caloric load. KE, which are made up of a BHB ester linked to butanediol, are next metabolized by the liver. KE can increased blood ketones above 1 mM, but their use is limited by gastric discomfort at high doses. D-BHB is directly absorbed into circulation and distributed throughout the body [127]. Pimental-Suarez et al. investigated the safety and tolerability of D-BHB, a bioidentical and salt-free ketone supplement. Healthy adults aged 18 to 69 years received 10 g of D-BHB diluted in water once daily for 28 days. Venous blood gas analysis was conducted at the baseline and every two weeks, while urinalysis was performed to monitor urinary ketone levels. The most common adverse symptom is gastrointestinal (GI) discomfort (2.6%); however it is tolerable without severe adverse reactions. The vital signs and blood work acid-base parameters, as well as electrolytes, were in the range of normal [128]. The profile of adverse events from D-BHB supplementation was similar to that reported by Gregor et al., in whose study gastrointestinal symptoms, particularly gastric reflux (33%), were the most common, while other side effects were mild to moderate. In their study, a dose equivalent to 170 mg/kg for a 70 kg adult was used. D-BHB supplementation mildly increased blood ketone levels and suppressed lipolysis. Overall, the supplement was considered safe and tolerable [129].

Thomsen et al. demonstrated that BHB infusion during LPS-induced acute inflammation increased circulating BHB levels and reduced whole-body protein degradation, as evidenced by decreased phenylalanine to tyrosine conversion and lower net phenylalanine release from the forearm. In skeletal muscle, BHB enhanced elF2α phosphorylation and tended to reduce S6 kinase phosphorylation, through an mTOR-independent pathway. These findings suggest that BHB exerts significant anti-catabolic effects by preserving muscle protein during acute inflammatory stress [130].

Given its ability to alleviate muscle wasting, BHB supplementation could be explored for treating sarcopenia, critical illness, metabolic disorders, inflammatory disease, and even aiding athletic recovery. Future research should focus on optimizing dosage, evaluating long-term effects, and exploring potential synergies with exercise or other metabolic modulators to maximize therapeutic benefits. In recent clinical trials, the effects of BHB supplementation as an intervention for several diseases have been investigated, although the results are not fully available yet (Table 4).

## 6. Conclusions

The chemical stability of BHB, compared to other ketone bodies such as acetoacetate and acetone, allows it to persist longer in the blood stream and remain readily available for energy metabolism and signaling functions. Beyond serving as an alternative energy source during glucose depletion, such as fasting, CR, or starvation, BHB also plays multiple roles as a signaling molecule by regulating gene expression, the sympathetic nervous system, lipid metabolism, and inflammation [131]. BHB inhibits class I HDACs, leading to increase histone acetylation which in turn promotes the expression of genes that mitigate oxidative stress, suppress the NLRP3 inflammasome, and regulate GPCRs. Through the activation of HCAR2, BHB also can attenuate inflammatory responses in various cell types, including macrophages and endothelial cells, thereby potentially reducing the risk of chronic inflammatory diseases [132,133]. BHB’s role in muscle–brain crosstalk highlights its therapeutic potential in conditions such as sarcopenia, metabolic disorders, and neurodegenerative diseases by enhancing mitochondrial function, reducing inflammation, and supporting muscle maintenance.

Additionally, BHB influences liver, adipose tissue, and immune-system function, suggesting broader implications in metabolic disorders, aging, and cancer. Despite these promising findings, further research is needed to elucidate optimal dosing strategies, long-term effects, and BHB’s translational potential in clinical settings.

Understanding the mechanisms of BHB in disease contexts will pave the way for its development as a novel therapeutic agent across multiple organ systems.

## Figures and Tables

**Table 2:** Summary of in vitro and preclinical findings on BHB in cancer models.

Cancer type	Model	Key Effects of BHB	Mechanism	Reference
Colorectal	In vivo: CAC mice and organoids	↓ Tumor growth, ↑ Expression of Hopx	HCAR2	[82]
Lung cancer	In vitro: A549	↑ Apoptosis, ↑ ROS, ↑ Mitochondrial dysfunction	CD133, CD44, and SOX-9 downregulation	[83]
Glioma	In vitro: Glioma U251 cells	↓ Angiogenesis, Invasion of glioma cells, ↓ EMT process, ↓ Stemness of glioma	GFβ/PI3K/Akt/GSK- 3β and Wnt5a/β-catenin/Snail pathways	[84]
Colon cancer	In vitro: 5FU treated SW480	↑ Expression of genes associated with stemness and mitochondrial biogenesis, ↓ Expression genes related to glycolytic program and differentiation	5FU-treated SW480 have the ability to use metabolic energy source of BHB, as well as stemness features also increasing to survive	[85]

Note: Most of the findings listed are based on in vitro or ex vivo models and may not directly reflect in vivo carcinogenic processes.

**Table 3:** Summary of the physiological and cellular effects of BHB and ketogenic intervention across experimental contexts.

Study Type	Model/System Used	Intervention or Focus	Main Findings	References
Clinical	Adults at risk of T2D (Study 1)	Ex vivo BHB treatment of LPS-stimulated leukocytes	↓ IL-1β, and TNF-α, IL-6; ↑ IL-10 and IL-1RA	[98]
	T2D patients (Study 2)	Study single oral dose of ketone monoester (KME)	↑ plasma IL-10 at 90 min post a single KME dose, coinciding with peak blood BHB in T2D	
	T2D patients (Study 3)	14 days of KME supplementation (3 times a day) + ex vivo BHB on leukocyte	No change in plasma cytokines or immune cells; ex vivo effects still observed in glucose- and time-dependent manner	
Clinical	Obese patients with advanced diabetic nephropathy	12-week very low-calorie ketogenic diet + exercise	↓ weight (12%), ↓ albuminuria (reduction 36%, ns), ↓ serum creatinine and cystatin C	[124]
In vivo	6-ODHA-induced Parkinson’s disease in rats	Strict ketogenic diet (7 weeks total)	No neuroprotection despite hyperketonemia: slight behavioral improvement	[125]
In vivo and in vitro	HK-2 cells, C57 BKS db/db mice, diabetic kidney tissue from patients	BDH1 overexpression, BHB supplementation, ketogenic diet	BDH1 is downregulated in DKD; its overexpression or BHB/KD reverses oxidative stress, inflammation, fibrosis via NRF2 activation	[126]
In vitro and in vivo	HK-2 cells and db/db mice with diabetic nephrophaty	KD, 3-OHB treatment	3-OHB delayed DN progression; ↑ Autophagy (LC3-II, beclin), ↑ AMPK/NRF2, ↓ ROS; Reduced albuminuria and mesangial expansion cell	[97]

**Table 4:** Summary of clinical trials investigating beta-hydroxybutyrate (BHB) supplementation in various conditions. Data include intervention type, target condition, participant group, recruitment status, clinical trial registration number (ClinicalTrials.gov), and study phase.

Intervention	Disease/Effect	Subject	Status	Clinical Trial No	Phase
Beta Hydroxybutyra Ester (KetoneaID ke4)	ALS	Patients with ALS	Recruiting	NCT04820478	N.A.
Dietary Supplement: 3-OHB (Oral)	T2D, Ketosis	Patients with T2D	Completed	NCT05263401	N.A.
Dietary Supplement: BHB	Ketosis, Ketonemia	Healthy individuals	Completed	NCT05980858	N.A.
Dietary Supplement: D-BHB	Ketosis	Healthy individuals	Completed	NCT04881526	N.A.
BHB supplementation (Oral)	Chron’s Disease, IBD	Patients with IBS	Recruiting	NCT06351124	Phase 1 and 2
Dietary Supplement: KME vs. BHB vs. 1,3-Butanediol	Ketosis	Healthy individuals	Completed	NCT05273411	N.A.
Dietary Supplement: 3-OHB salt (NaCl)	Healthy, Incretin Effect, Ketosis	Healthy individuals	Completed	NCT03935841	N.A.
Dietary Supplement: KME vs. Control Supplement: carbohydrate-fat placebo (fructose, corn and canola oil 50:50 ratio)	Preservation of muscle mass and function (i.e., protein synthesis, insulin sensitivity, mitochondrial function)	Healthy individuals	Completed	NCT05679596	N.A.
Dietary Supplement: 3-OHB + Whey vs. Whey	Maintenance of muscle mass in catabolic status (Healthy individuals with LPS injection)	Healthy individuals	Completed	NCT04064268	N.A.
Dietary Supplement: KME vs. Placebo supplement	SCD	Adults with SCD (55 to 75 years old)	Not yet recruiting	NCT06588946	Phase 2

ALS, amyotrophic lateral sclerosis; BHB, beta-hydroxybutyrate; 3-OHB, 3-hydroxybutyrate; DM, diabetes mellitus; IBD, inflammatory bowel disease; KME, ketone monoester ((R)-3-hydroxybutyl (R)-3-hydroxybutyrate); LPS, lipopolysaccharide; SCD, subjective cognitive decline. N.A., not available.

## Data Availability

Not applicable.
